# The Use of E-Cigarettes among High School Students in Poland Is Associated with Health Locus of Control but Not with Health Literacy: A Cross-Sectional Study

**DOI:** 10.3390/toxics10010041

**Published:** 2022-01-17

**Authors:** Mariusz Duplaga, Marcin Grysztar

**Affiliations:** Department of Health Promotion and e-Health, Institute of Public Health, Faculty of Health Sciences, Jagiellonian University Medical College, Skawinska Str. 8, 31-066 Krakow, Poland; marcin.grysztar@uj.edu.pl

**Keywords:** electronic cigarettes, e-cigarettes, high school students, adolescents, health literacy, health locus of control, European Health Literacy Survey questionnaire, pen and pencil interviewing, logistic regression

## Abstract

Since their introduction, the use of electronic cigarettes has increased considerably in the population and among adolescents. Determinants of smoking conventional cigarettes were thoroughly studied in various social groups. However, we know less about the predictors of the use of e-cigarettes in younger generations. The main aim of this study was the assessment of the factors associated with the use of electronic cigarettes among high school students. Specifically, the roles of health literacy (HL) and health locus of control (HLC) were addressed. The analysis was based on the data from a ‘pen-and-pencil’ survey performed in a large sample of 2223 high school students from southern Poland. The tools used in the survey encompassed 133 items, including a 47-item European Health Literacy Survey questionnaire, an 18-item Multidimensional Health Locus of Control Scale, and a set of questions asking about the health behaviors, and sociodemographic and economic characteristics of respondents. In the study sample, 47.5% of the respondents had used e-cigarettes in the past, and 18.6% had used them in the last month. HL was not significantly associated with dependent variables reflecting the use of e-cigarettes. Two types of external HLC were associated with using e-cigarettes in the past, and ‘Chance’ HLC (CHLC) was also associated with their use in the last month. Males, students of schools providing vocational training, and students declaring more Internet use during the week showed a higher likelihood of ever using e-cigarettes or using them in the last month. Students smoking conventional cigarettes were also more prone to use e-cigarettes. To sum up, it was an unexpected result that HL is not associated with the use of e-cigarettes. A greater likelihood of using e-cigarettes was positively associated with higher CHLC scores, as in the case of smoking traditional cigarettes.

## 1. Introduction

The use of e-cigarettes by youth has increased considerably in the last decade [1]. According to Fadus et al. [2], the use of e-cigarettes among youth has increased from 1.5% in 2011 to 20.8% in 2018. The growing use of e-cigarettes among this population has been explained by various factors, including advertising exposure, the availability of flavors attractive to youth, the introduction of easily concealable devices with high nicotine content, their user-friendly function, and their ability to be used discreetly in places where smoking is prohibited [2,3].

A USA study has shown that a significant increase in e-cigarettes sales was accompanied by only a small decrease in conventional cigarette sales during the period of 2011–2015 [4]. Although e-cigarettes were marketed as a smoking cessation means, the results of a large study showed that there has been only a marginal decline of regular smoking among youth since 2010 when e-cigarettes emerged on the market [5]. Fadus et al. suggested that, on the contrary, the use of e-cigarettes may have a “gateway” effect for combustible cigarettes and cannabis use [2]. The observations from Poland tend to confirm these claims. Smith et al. have found that exclusive use of e-cigarettes among adolescents 15–19 years old in Poland increased from 2.0% in 2010–2011 to 11% in 2015–2016 [6]. However, dual-use also increased, from 4% in 2010–2011 to 23% in 2015–2016 [6]. Interestingly, as many as 76% of dual users confirmed (in the study from 2015–2016) that they had used cigarettes before trying e-cigarettes. According to the cross-sectional study performed in two waves between 2014 and 2018 by Kaleta and Polanska [7], the percentage of secondary school girls using e-cigarettes increased from 20.7% to 31.7%. Furthermore, the smoking of traditional cigarettes has been stable in the period covered by the study and has remained on the level from 25.1% to 27.9%. There was a significant increase of dual use among older boys from 45.7% in 2014–2015 to 56.8% in 2017–2018 [7]. Another study, performed in Poland within the Global Youth Tobacco Survey among 11–17 year old youth in 2016, revealed that 31.5% of boys and 21.8% of girls were current e-cigarette users and 21.8% of boys and 19.9% of girls smoked traditional cigarettes [8]. Dual uses made up 14% of the respondents in this study group. All these reports indicate that the use of e-cigarettes among adolescents has become an urgent public health issue requiring adequate attention.

According to Wallston [9], health locus of control (HLC) reflects the degree to which individuals believe that their health status remains under their own control or is influenced by factors external to themselves, e.g., other people, fate, chance, or some undefined ‘higher power’. In 1978, Wallston et al. developed the Multidimensional Health Locus of Control (MHLC) scale measuring three dimensions of HLC, internal (IHLC) and two external called ‘Powerful Other’ (OHLC) and “Chance” HLC (CHLC) [10]. People with higher IHLC are convinced that they can control their health through appropriate behaviors. Higher external HLC shows that a person is more inclined to attribute their health status either to other people or to chance factors. It has been demonstrated in various populations that the MHLC score may be associated with health behaviors [11,12,13,14], quality of life [15], and self-assessed health [16]. Many authors have also reported a significant relationship between HLC and smoking, indicating that high CHLC predicted active smoking or resuming smoking after control programs [17,18,19,20,21,22,23]. It remains in line with the theory of HLC that explains that people with high CHLC believe that health is independent of their personal health behaviors. 

In 1982, Clarke et al. reported that adolescents with an external locus of control were the group at the greatest risk that they would start smoking early, smoke at a high frequency, and continue smoking behavior [24]. Eiser et al. observed, among a large group of school students 11–16 years old, that smokers, compared to non-smokers, showed lower OHLC and IHLC and higher belief in the importance of a “chance” influence on their health outcomes [25]. Many more recent studies confirmed a significant association between external HLC and smoking habits among youths and young adults [26,27,28,29]. Unfortunately, the relationship between HLC and the proclivity towards the use of e-cigarettes has not been reported on yet.

There are many definitions of health literacy (HL). According to the World Health Organization, HL may be perceived as the cognitive and social skills resulting in individuals’ motivation and ability to access, understand, and use information to promote health [30]. The level of HL may be assessed with general-purpose or domain-specific instruments. Currently, the questionnaire developed within the European Health Literacy Survey (EHLS) is one of the most popular tools used to assess general HL in population studies [31]. Its basic form consists of 47 items (HLS-EU-Q47) evaluating the abilities to access, understand, appraise, and apply health information in the domains of health promotion, disease prevention, and health care [31]. It was evidenced that HL is one of the key factors associated with health behaviors, utilization of health services, and the ability to communicate with health care providers [32,33,34]. Although the association between HL and smoking traditional cigarettes has frequently been studied, reports on e-cigarettes are relatively rare. 

The survey performed in several European countries by the EHLS project team revealed that HL is significantly associated with smoking and other health behaviors. However, the study conducted among the Polish population in 2016 did not show a significant association between HL and smoking [35]. A recent survey carried out by Clifford et al. revealed that respondents with higher levels of oral HL were less likely to be current dual users of e-cigarettes and conventional cigarettes [36]. Interestingly, these authors did not find a significant association between written HL and either the smoking of traditional cigarettes or the use of e-cigarettes. As for the adolescents, the study performed in the Netherlands, Germany, and Finland among adolescents 14–16 years old revealed that HL was not associated with smoking. Still, it was positively related to beliefs about the consequences of smoking [37]. The fact that the use of e-cigarettes became an issue in public health seems to be supported by the recent development of the e-Cigarette Use Health Literacy scale [38].

The main aim of this study was to assess the association of socio-demographic and economic factors, HL and HLC, with the use of e-cigarettes among high school students from southern Poland. Furthermore, the relationship between the use of e-cigarettes and conventional cigarettes among this population was analyzed.

## 2. Materials and Methods

### 2.1. Survey

The study reports the analysis of data obtained from a sample of high school students from a district (Malopolska Voivodship) in southern Poland. The survey was performed with the ‘pen-and-paper’ technique. The respondents were selected as the result of cluster two-stage random sampling. In the first stage, twenty high schools located in the Malopolska Voivodship were randomly selected from the repository of schools maintained by the Board of Education. The directors of these schools were invited to the study; nine responded positively. In each of these schools, 5–10 classes were randomly selected for the survey, considering grades and profiles. The parents of students attending selected classes were informed about the study aims and procedures. Parents of students younger than 18 years old were asked for consent to include their child in the survey. All students attending the selected classes were informed about the study and asked for their informed consent. Data were collected in the period from September to October 2017. 

The study was conducted after receiving consent from the Bioethical Committee of Jagiellonian University issued on 25 September 2014 (KBET/193/B/2014). 

The questionnaire used in the survey encompassed 130 individual items, including questions exploring the health behaviors and socioeconomic status of respondents, the questionnaire developed within the EHLS project consisting of 47 items (HLS-EU-Q47) [39], and the MHLC scale composed of 18 items [40]. Only the Polish version of the questionnaire was applied. The items used in the analysis in this paper (apart from earlier validated tools such as HLS-EU-Q47 and MHLC scale) have been translated to English and are available in the Supplementary Materials file.

### 2.2. Dependent and Independent Variables

Two dichotomous variables reflecting the use of electronic cigarettes were applied as dependent variables in univariate and multivariate logistic regression models. They were derived from items asking if respondents had ever used e-cigarettes (yes coded as ‘1’ vs. no coded as ‘0’) and about their use in the last month (yes coded as ‘1’ vs. no coded as ‘0’). 

In the univariate and multivariate logistic regression models, the following independent variables were applied:

- Sociodemographic variables: gender, attended class in school (treated as a proxy of respondent’s age), place of residence, marital status of parents, the levels of education of the mother and farther, the number of siblings, the category of school (including vocational training or not);
- Variables reflecting the financial situation of the respondent’s family: receiving external help, self-assessment of family economic status, and monthly expenses for a mobile phone;
- The use of information technology—variables indicating the time spent on the Internet per week;
- Health literacy score based on the HLS-EU-Q47.

The HL score was established according to the guidelines from the EHLS project team; responses to each item included in the HLS-EU-Q47 were transformed to individual scores from 1 to 4 [39]. If the respondent was not decided or missed the response for a particular item, it was treated as a missing value. A percentage of missing values surpassing 20% in the HLS-EU-Q47 questionnaire precluded calculating the general score for the respondent.

- Three subscores of the MHLC scale measuring IHLC, OHLC, and CHLC.

As recommended by the authors of the scale [10,40], the responses obtained according to the Likert scale from strongly disagree to strongly agree were assigned numerical values from 1 to 6. Subscales reflecting IHLC, OHLC, and CHLC were calculated as sums of six relevant items. As a result, subscales’ scores could range from 6 to 36. Wallston et al. [10] explained that each type of HLC is not mutually exclusive; therefore, all three scores were applied in multivariate regression modeling.

The variables showing ‘ever used’ and ‘used in the last month’ of conventional cigarettes were utilized to analyze their association with the use of electronic cigarettes.

### 2.3. Statistical Analysis

The software package IBM SPSS Statistics v.26 (IBM Corp., Armonk, NY, USA) was utilized for statistical assessment. Relative and absolute frequencies were used to describe categorical variables and the mean and standard deviation (SD) for continuous numerical variables.

The roles of potential predictors of the use of e-cigarettes were assessed with univariate and multivariate logistic regression models. Each model was characterized with the Hosmer–Lemeshow test. Furthermore, for all regression models, Nagelkerke R2 was also established. In the Result section, the effect of independent variables was presented as the odds ratio (OR), 95% confidence interval (95% CI), and *p*-values in the case of univariate models and adjusted OR (aOR), 95% CI and *p*-value for multivariate models. Statistical significance was established as *p* < 0.05.

## 3. Results

### 3.1. Characteristics of the Study Group

The number of questionnaires included in the final analysis after quality control was 2223 (response rate 95.4%). Girls made 66.3% (*n* = 1457) of the study group, students of high schools providing general education—82.3% (*n* = 1829). The average age of the study participant was 17.01 years (SD = 0.97). Students attending I class were 37.0% (*n* = 1457), II class—28.8% (*n* = 630) and III or IV class—34.2% (748). The average HL score was 34.76% (SD = 6.13), IHLC 25.04 (SD = 4.59), OHLC—20.60 (SD = 4.99) and CHLC—19.89 (SD = 5.29). Detailed characteristics of the study group have already been published [41].

### 3.2. The Use of E-Cigarettes and Smoking of Conventional Cigarettes

It was shown that the use of e-cigarettes in the past and the use in the last 30 days are significantly associated with smoking traditional cigarettes in the past and smoking in the previous 30 days (Table 1). Among respondents who have ever used e-cigarettes, 87.6% (*n* = 923) had smoked traditional cigarettes in the past. The percentage of respondents who had ever smoked traditional cigarettes among those who had never used e-cigarettes was only 22.0% (*n* = 249) (Fisher exact test, *p* < 0.001). The percentages of the respondents who had smoked traditional cigarettes in the last 30 days among those who had ever used and never used e-cigarettes were 55.4% (586) and 8.8% (*n* = 102), respectively (Fisher exact test, <0.001). Furthermore, the percentage of respondents who had ever smoked traditional cigarettes, among those who had used and had not used e-cigarettes in the last 30 days, was 92.7% (*n* = 381) and 44.6% (*n* = 795), respectively (Fisher exact test, *p* < 0.001). Finally, respondents who had smoked traditional cigarettes in the last 30 days, among those who had or had not used e-cigarettes in the previous 30 days, were 72.4% (*n* = 299) and 21.7% (*n* = 392), respectively (Fisher exact test, *p* < 0.001). 

### 3.3. Predictors of Having Ever Used E-Cigarettes in the Past

High school students who had ever used e-cigarettes in the past were 47.5% (*n* = 1057). Univariate logistic regression revealed that significant predictors of having ever used e-cigarettes in the past included gender, the type of school, attended class (year of high school) at the moment of the survey, size of the accommodation, the number of siblings, marital status of parents, the expenses on mobile phone, the weekly duration of Internet use, the number of books at home, PHLC, and CHLC (Table 2). Boys were more likely to use e-cigarettes than girls (OR, 95%CI: 1.28, 1.07–1.52). Students of vocational education schools were as much as 2.28 times more likely to use e-cigarettes than those from general education schools (OR, 95%CI: 2.28, 1.82–2.86). Furthermore, the oldest students were more likely than the youngest (attending III or IV class in comparison to attending I class, OR, 95% CI: 1.47, 1.20–1.80), those whose parents were divorced or separated than those whose parents were married (OR, 95% CI: 1.45, 1.10–1.92), and those spending the most on the mobile phone were more likely than those spending the least (OR, 95% CI: 2.11, 1.39–3.22), to use e-cigarettes. More frequent use of the Internet has also been associated with a higher likelihood of using e-cigarettes (OR, 95% CI: 2.20, 1.56–3.09). Higher CHLC was also a predictor of more frequent use of e-cigarettes (OR, 95%CI: 1.21, 1.10–1.33). Having more than two siblings rather than no siblings (OR, 95% CI: 0.65, 0.46–0.91), living in the largest home rather than in the smallest (OR, 95% CI: 0.74, 0.55–0.99), and having at home more than 50 books rather than having less than 25 books (OR, 95% CI for comparison between those having the smallest and greatest number of books: 0.50, 0.34–0.72) was associated with less frequent use of e-cigarettes. A higher rather than a lower OHLC score was also related to less frequent use (OR, 95% CI: 0.86, 0.78–0.95). 

In the multivariate regression model, a significant association was maintained only for gender, type of school, attended class, mobile phone expenses, Internet use duration, OHLC, and CHLC (Table 2). The association between the use of e-cigarettes and duration of Internet use became significant for all but one category of longer Internet use in the multivariate model. 

### 3.4. Predictors of Regular Use of E-cigarettes

18.6% (*n* = 413) of the study participants had used e-cigarettes in the last month. The regression model developed for the use of e-cigarettes in the previous month revealed similar relationships as for their ever having been used in the past for gender, type of school, marital status of parents, the number of books at home, the duration of weekly Internet use, and CHLC (Table 3). However, no significant association has been confirmed between use of e-cigarettes in the last 30 days and the year in high school, the number of siblings, the size of home, and OHLC. Interestingly, new relationships have been revealed. Having a mother with higher levels of attained education rather than the lowest was associated with more frequent use of e-cigarettes (OR, 95% CI: 1.47, 1.10–1.98 and 1.60, 1.19–2.15, respectively). Furthermore, inhabitants of more populated urban areas were more prone to use e-cigarettes than those living in rural areas (OR, 95% CI: 1.40, 1.07–1.84). In the multivariate regression model, the significant association of using e-cigarettes in the previous 30 days was maintained for the same independent variables as in univariate regression models (Table 3). 

## 4. Discussion

In our study, we have analyzed the data from a survey on a large sample of 2223 students of high schools located in urban and rural areas of a voivodship in southern Poland. We have addressed the problem of the use of e-cigarettes within a broader survey focused on the health behaviors of Polish adolescents and their relationships with selected potential predictors. We have assumed that HLC may serve as a construct to explain, to some extent, the mechanisms leading to the use of e-cigarettes in this group. The decision to include HL assessment in our survey was dictated by our attempt to measure the level of HL in the adolescent population (as this has not been done before) and understand if adequate HL protects against potentially risky health behaviors in this group. 

We have shown that the likelihood that respondents have ever used e-cigarettes was higher among boys than girls, among students of schools providing vocational training than only general education, among older students than younger students, among respondents who spend the most on mobile phones than those paying the least as well as among the respondents using the Internet for the longest time per week than among those using it for the shortest time. The level of HL was not significantly associated with the likelihood of using e-cigarettes. Finally, it was significantly lower among persons with higher OHLC scores and higher among persons with greater CHLC. The multivariate logistic regression model has shown a similar pattern of interrelationships between the use of e-cigarettes in the last 30 days and gender, type of school, weekly duration of Internet use, and CHLC, but not with attended class, expenses on mobile phone and OHLC. We have also observed that recent use of e-cigarettes was significantly associated with the level of education attained by respondents’ mothers, place of residence, marital status, and the number of books at home. 

The higher prevalence of the use of electronic cigarettes among males than females has been reported in many previous studies [42,43,44,45,46]. Additionally, older adolescents in the studied groups showed higher use of e-cigarettes [42,47,48]. Vuolo et al. reported a higher likelihood of using e-cigarettes among 17 years old adolescents from families with only one parent or whose parents were divorced [44]. Contrary to our findings, the study published recently by Janik-Koncewicz et al. showed that the use of e-cigarettes among Polish students was higher among those living in rural rather than urban areas [8]. In turn, Vuolo et al. found that youths from larger cities were more prone to use e-cigarettes [44]. A higher incidence of vocational students using e-cigarettes has been seen by Surís et al. [46].

We have used the expenses on mobile phones as an indicator of the economic status of the family of the respondent. The respondents who spent the most on their mobile phones have exhibited a higher likelihood of using e-cigarettes than those paying the least. The relationships with other variables that could be treated as indicators of economic status of a given respondent and the use of e-cigarettes have also been observed by other authors [48].

In our study, we have found that using e-cigarettes is significantly associated with the smoking of conventional cigarettes. The percentage of respondents who smoked cigarettes among the recent users of e-cigarettes was about 72%. In the study performed by Janik-Koncewicz et al., this percentage was 55% [8]. In turn, we have found that among current smokers of conventional cigarettes, 43% also used e-cigarettes, and in the study of Janik-Koncewicz et al., 69% did [8]. Many other authors have studied and confirmed the relationship between smoking conventional cigarettes and using e-cigarettes [42,44,45].

More Internet use during the week was associated with higher odds of using e-cigarettes among Polish adolescents. Interestingly, Lee et al. observed in South Korean youth that the respondents not using the Internet were more prone to use all types of tobacco products and e-cigarettes [49]. Other authors have suggested a significant association between the use of electronic cigarettes and the use of the Internet or information-seeking behaviors online [50,51]. One possible explanation for the relationship between more intensive Internet use and online information search behaviors and the use of e-cigarettes is the positive sentiment toward e-cigarettes on the Internet and especially social media [52].

The importance of HLC as a predictor of the smoking of conventional cigarettes among the general population and adolescents has been reported earlier by many authors [21,22,23,24,25,26,27,29]. Usually, they confirmed that higher CHLC was related to the higher likelihood of smoking. Our study is probably one of the first to confirm that CHLC is also consistently associated with the likelihood of using e-cigarettes either ever in the past or during the last 30 days before the survey. This finding may suggest that, to some extent, similar mechanisms, as in the case of conventional cigarettes, are responsible for initiating and maintaining the use of e-cigarettes in youths.

There was no significant relationship between HL and the use of e-cigarettes in Polish high school students. Previous studies analyzing the association between the level of HL and health behaviors have yielded unambiguous results. Sørensen et al. reported that among the population 15–75 years old, HL, as measured with HLS–EU–Q47, has been significantly associated with smoking conventional cigarettes, but the correlation was rather low [31]. The study performed in 2016 among the adult Polish population with a short, 16-item version of the HLS–EU questionnaire did not show a significant association between HL and smoking [35]. Only a few studies indicate a relationship between HL and the use of e-cigarettes. Recently, Clifford et al. found no significant relationship between HL and conventional cigarettes, e-cigarettes, or dual-use among the large sample of respondents recruited for the 2016 Behavioral Risk Factor Surveillance System [36]. It seems that our study is the first to report the results of the analysis of the association between the levels of HL and the use of electronic cigarettes by adolescents. The lack of a significant relationship between HL and the use of e-cigarettes among Polish adolescents may be an important indication that interventions increasing health-related knowledge and skills, especially those provided at schools, should put more emphasis on the aspects related to smoking and the use of e-cigarettes. Currently, these aspects are not adequately covered by health education focused on younger generation. 

Due to its limitations, in our study, we have not been able to address many other determinants reported by other authors, including opinions about the social acceptability of e-cigarettes and the sensory experience [53], daily cannabis use and frequent alcohol use [44], exposure to secondhand smoking in various places [45] or early sexual experience [44].

### Limitations

Our study was aimed at the assessment of many types of health behaviors. Therefore, we have included only a limited set of items asking about using e-cigarettes. Due to the broad scope of potential determinants addressed in the survey, our analysis covers variables reflecting the socio-demographic and financial status of respondents and HLC and HL.

However, it should be noted that the study reported here has not addressed many potential factors that can influence the acceptability and use of e-cigarettes. In their review, Trucco et al. mention several attributes increasing the appeal of e-cigarettes to youth, such as flavor variety, device modifiability, the ability to perform tricks, and concealment from authority figures [54]. It is also well known that adolescents have high positive expectations about e-cigarettes associated with personal enjoyment, social benefits, and perceived safety [54].

The number of students who refused to participate in the survey was 107 (4.56% of all the approached students). It seems that this fraction should not considerably influence the results reported in the paper. The questionnaire applied in the survey consisted of about 130 individual items and this could result in a lower quality of response. However, we have not observed a high number of missing responses. In the case of the HL score, we were not able to calculate it for only 6.3% of the returned questionnaires, partially due to the fact that some of the respondents selected the response option ‘not applicable’. The number of missing responses in the HLC scale was very low, not surpassing 1.0% of the questionnaires.

This study was carried out in only one district of southern Poland, and it would be risky to extend the obtained results to the whole Polish population of adolescents. On the other hand, good representation of urban and rural communities and various types of included schools allows the assessment of multiple factors that could influence the use of e-cigarettes.

The analysis reported here has been performed on data collected in 2017, and the obtained results should be treated cautiously as the trends in the use of e-cigarettes and other emerging nicotine delivery products are changing quickly.

## 5. Conclusions

Adolescents have become one of the main groups of users of new nicotine delivery products, including e-cigarettes. Contrary to marketing slogans, e-cigarettes can hardly be treated as a tool used to limit the use of conventional cigarettes among this population. Similar to other reports, our study clearly shows that the users of e-cigarettes are also frequently smokers of traditional cigarettes. We have found that among the sociodemographic factors, gender is the main predictor of the use of electronic cigarettes. Some effects, especially on the recent use of e-cigarettes, have also been found for the place of residence, marital status of parents, and the level of education of the mothers but not the fathers of respondents. Unexpectedly, respondents whose mothers have attained higher levels of education showed a more increased risk of using e-cigarettes. It also seems that respondents living in urban areas are more prone to use e-cigarettes than inhabitants of rural areas. This study showed that intensive Internet users show a higher proclivity toward electronic cigarettes than those using the Internet for a shorter time weekly. Finally, HL did not affect the use of e-cigarettes, but CHLC was a consistent predictor.

The results of this study also suggest directions for future research. It should be explained why general HL is not associated with a cautious approach to smoking e-cigarettes. Furthermore, health educators should be aware that there are strong interrelations between smoking traditional cigarettes and the use of e-cigarettes. This implies that e-cigarettes cannot be treated as a means of preventing the initiation of smoking cigarettes or a contingency measure, at least among adolescents. 

In practical terms, health promotion interventions focused on improving the HL of children and adolescents should strive for better understanding of the potential adverse effects of e-cigarettes as well as the relationships between their use and smoking conventional cigarettes. It also seems that the use of e-cigarettes by adolescents has become an important public health issue. Still, neither the mechanisms nor consequences of this phenomenon are sufficiently understood or adequately addressed in public health policies.

## Figures and Tables

**Table 1:** Association between using e-cigarettes and smoking conventional cigarettes.

Variables		Using E-Cigarettes					
		Ever Used E-Cigarettes			Used E-Cigarettes in the Last 30 Days		
Smoking Conventional Cigarettes		no % (*n*)	yes % (*n*)	*p*	no % (*n*)	yes % (*n*)	*p*
ever smoked	no	78.0 (885)	12.4 (131)	<0.001	55.4 (989)	73 (30)	<0.001
	yes	22.0 (249)	87.6 (923)		44.6 (795)	92.7 (381)	
smoked in last 30 days	no	91.2 (1051)	44.6 (471)	<0.001	78.3 (1418)	27.6 (114)	<0.001
	yes	8.8 (102)	55.4 (586)		21.7 (392)	72.4 (299)	

Abbreviations: *p*—Fisher exact test.

**Table 2:** Uni- and multivariate logistic regression models for the use of e-cigarettes in the past.

Variables	Categories	The Use of E–Cigarettes Ever in Past			
		OR (95% CI)	*p*	aOR (95% CI)	*p*
Gender	Female *				
	male	1.28 (1.07–1.52)	0.007	1.44 (1.17–1.79)	0.001
Type of school	general education				
	vocational training	2.28 (1.82–2.86)	<0.001	2.16 (1.63–2.86)	<0.001
Attended class in school	class I *				
	class II	1.16 (0.94–1.44)	0.155	1.17 (0.91–1.50)	0.216
	class III or IV	1.47 (1.2–1.8)	< 0.001	1.45 (1.15–1.83)	0.002
Mother’s level of education	lower than secondary *				
	secondary	1.21 (0.97–1.5)	0.090	1.18 (0.90–1.55)	0.233
	university	1.06 (0.86–1.32)	0.574	1.20 (0.88–1.62)	0.246
Father’s level of education	lower than secondary *				
	secondary	1.08 (0.88–1.31)	0.455	0.95 (0.74–1.22)	0.702
	university	0.84 (0.68–1.03)	0.101	0.83 (0.61–1.12)	0.219
Place of residence	Rural *				
	urban ≤10,000	1.15 (0.8–1.65)	0.447	1.00 (0.65–1.53)	0.992
	urban > 10,000 to 200,000	1.13 (0.91–1.41)	0.278	0.99 (0.75–1.30)	0.919
	urban >200,000	0.98 (0.79–1.20)	0.825	0.82 (0.61–1.11)	0.199
Size of home	≤50 m^2^ *				
	>50 m^2^–70 m^2^	0.85 (0.6–1.20)	0.362	0.97 (0.65–1.46)	0.883
	>70 m^2^–90 m^2^	0.72 (0.5–1.03)	0.074	0.73 (0.47–1.13)	0.162
	>90 m^2^	0.74 (0.55–0.99)	0.041	0.79 (0.54–1.15)	0.215
Number of siblings	0 *				
	1	0.95 (0.77–1.16)	0.620	1.09 (0.85–1.40)	0.495
	2	0.9 (0.70–1.16)	0.421	1.20 (0.87–1.64)	0.271
	>2	0.65 (0.46–0.91)	0.011	0.76 (0.50–1.17)	0.210
Marital status	Married *				
	divorced or separated	1.45 (1.10–1.92)	0.009	1.37 (0.97–1.92)	0.072
	one or both parents dead	1.04 (0.65–1.67)	0.875	1.06 (0.60–1.89)	0.842
External help	No *				
	yes	1.16 (0.98–1.38)	0.084	1.03 (0.83–1.28)	0.792
The self–assessed financial situation of the family	worse than good *				
	good	1.01 (0.79–1.30)	0.919	1.06 (0.79–1.44)	0.683
	very good	1.04 (0.79–1.36)	0.786	1.19 (0.85–1.66)	0.308
Monthly expenses on mobile phone	≤5 PLN *				
	>5–10 PLN	0.73 (0.43–1.22)	0.223	0.96 (0.53–1.75)	0.894
	>10–30 PLN	1.13 (0.76–1.70)	0.543	1.37 (0.85–2.20)	0.202
	>30–50 PLN	1.39 (0.93–2.08)	0.111	1.59 (0.99–2.56)	0.056
	>50 PLN	2.11 (1.39–3.22)	<0.001	2.34 (1.42–3.86)	0.001
Books at home	≤25 *				
	26–50	0.75 (0.55–1.02)	0.067	0.88 (0.61–1.27)	0.502
	51–100	0.74 (0.55–0.99)	0.041	0.81 (0.58–1.14)	0.230
	101–500	0.66 (0.50–0.87)	0.003	0.73 (0.52–1.02)	0.067
	>500	0.50 (0.34–0.72)	<0.001	0.68 (0.44–1.06)	0.087
Duration of Internet use per week	not more than 2 h *				
	>2–7 h	1.48 (1.05–2.07)	0.025	1.67 (1.12–2.48)	0.012
	>7–14 h	1.36 (0.96–1.92)	0.083	1.63 (1.09–2.45)	0.018
	>14–21 h	1.21 (0.85–1.72)	0.299	1.35 (0.89–2.04)	0.158
	>21–35 h	1.37 (0.96–1.95)	0.085	1.52 (1.00–2.29)	0.048
	>35 h	2.2 (1.56–3.09)	<0.001	2.38 (1.59–3.57)	<0.001
HL		1.00 (0.99–1.02)	0.648	1.01 (0.99–1.02)	0.509
IHLC		1.00 (0.99–1.02)	0.700	1.01 (0.99–1.03)	0.329
OHLC		0.98 (0.96–0.99)	0.003	0.96 (0.94–0.98)	<0.001
CHLC		1.03 (1.02–1.05)	<0.001	1.05 (1.03–1.07)	<0.001

Abbreviations: *—referential category, OR—odds ratio, 95%CI—95% confidential interval, *p*—*p*-value for uni- and multivariate logistic regression model, HL—health literacy, IHLC—internal health locus of control, OHLC—‘powerful other’ health locus of control, CHLC—‘chance’ health locus of control.

**Table 3:** Uni- and multivariate logistic regression models for the use of cigarettes in the last month.

Variables	Categories	The Use of Cigarettes in the Last Month			
		OR (95% CI)	*p*	aOR (95% CI)	*p*
Gender	Female *				
	male	1.93 (1.55–2.40)	<0.001	1.96 (1.51–2.54)	<0.001
Type of school	general education *				
	vocational training	2.42 (1.89–3.10)	<0.001	2.34 (1.71–3.19)	<0.001
Attended class in school	class I *				
	class II	0.98 (0.75–1.28)	0.87	1.16 (0.85–1.58)	0.354
	class III or IV	0.95 (0.74–1.23)	0.711	1.00 (0.74–1.34)	0.981
Mother’s level of education	lower than secondary *				
	secondary	1.60 (1.19–2.14)	0.002	1.49 (1.04–2.13)	0.030
	university	1.47 (1.09–1.98)	0.011	1.72 (1.16–2.55)	0.007
Father’s level of education	lower than secondary *				
	secondary	1.12 (0.87–1.44)	0.385	0.88 (0.64–1.2)	0.413
	university	1.04 (0.79–1.36)	0.782	0.82 (0.56–1.19)	0.292
Place of residence	Rural *				
	urban ≤10,000	1.11 (0.69–1.78)	0.679	1.17 (0.67–2.04)	0.582
	urban >10,000 to 200,000	1.56 (1.19–2.06)	0.002	1.53 (1.09–2.14)	0.015
	urban >200,000	1.41 (1.08–1.83)	0.011	1.41 (0.98–2.03)	0.065
Size of home	≤50 m^2^ *				
	>50 m^2^–70 m^2^	1.08 (0.71–1.66)	0.706	1.33 (0.80–2.19)	0.271
	>70 m^2^–90 m^2^	0.99 (0.63–1.56)	0.96	1.36 (0.79–2.35)	0.261
	>90 m^2^	0.84 (0.58–1.21)	0.344	1.31 (0.82–2.11)	0.257
Number of siblings	0 *				
	1	1.12 (0.86–1.45)	0.391	1.26 (0.92–1.72)	0.143
	2	0.82 (0.58–1.14)	0.240	1.09 (0.72–1.64)	0.695
	>2	0.83 (0.53–1.29)	0.409	1.18 (0.68–2.04)	0.559
Marital status	Married *				
	divorced or separated	1.53 (1.11–2.11)	0.010	1.53 (1.04–2.26)	0.033
	one or both parents dead	0.84 (0.43–1.61)	0.591	0.64 (0.27–1.48)	0.297
External help	No *				
	yes	1.10 (0.88–1.37)	0.390	1.14 (0.87–1.51)	0.334
Self–assessed financial situation of the family	worse than good *				
	good	0.96 (0.70–1.33)	0.816	1.12 (0.76–1.65)	0.556
	very good	0.97 (0.69–1.37)	0.864	1.01 (0.66–1.55)	0.967
Monthly expenses on mobile phone	≤5 PLN *				
	>5–10 PLN	0.59 (0.31–1.14)	0.115	0.66 (0.31–1.41)	0.281
	>10–30 PLN	0.70 (0.43–1.15)	0.159	0.86 (0.49–1.53)	0.612
	>30–50 PLN	0.83 (0.51–1.35)	0.457	0.98 (0.56–1.73)	0.952
	>50 PLN	1.09 (0.66–1.80)	0.73	1.44 (0.80–2.58)	0.223
Books at home	≤25 *				
	26–50	0.81 (0.56–1.18)	0.277	1.00 (0.65–1.52)	0.987
	51–100	0.74 (0.52–1.04)	0.087	0.64 (0.43–0.96)	0.031
	101–500	0.63 (0.45–0.89)	0.008	0.58 (0.39–0.88)	0.009
	>500	0.55 (0.35–0.89)	0.014	0.6 (0.35–1.04)	0.070
Duration of Internet use in a week	not more than 2 h *				
	>2–7 h	1.14 (0.73–1.80)	0.560	1.05 (0.62–1.76)	0.865
	>7–14 h	1.36 (0.86–2.14)	0.187	1.55 (0.93–2.59)	0.094
	>14–21 h	0.99 (0.61–1.60)	0.972	0.90 (0.52–1.56)	0.714
	>21–35 h	1.01 (0.63–1.64)	0.952	1.02 (0.60–1.74)	0.945
	>35 h	1.88 (1.21–2.92)	0.005	1.8 (1.09–2.96)	0.021
HL		1.01 (0.99–1.02)	0.487	1.01 (0.99–1.03)	0.371
IHLC		1.00 (0.98–1.03)	0.841	1.00 (0.97–1.03)	0.981
OHLC		0.99 (0.97–1.01)	0.399	0.98 (0.95–1.00)	0.073
CHLC		1.03 (1.01–1.05)	0.003	1.04 (1.02–1.07)	0.001

Abbreviations: *—referential category, OR—odds ratio, 95%CI—95% confidential interval, *p*—*p*-value for uni- and multivariate logistic regression model, HL—health literacy, IHLC—internal health locus of control, OHLC—‘powerful other’ health locus of control, CHLC—‘chance’ health locus of control.

## Data Availability

The data that support the findings of this study are available on request from the corresponding author. The data are not publicly available due to privacy and ethical restrictions. The authors did not inform participants that public access to the survey data may be considered. Access to the data will be granted on a case-by–-case basis, following a justified request after receiving consent from the Bioethical Committee at Jagiellonian University.

## Supplementary Materials

The following are available online at https://www.mdpi.com/article/10.3390/toxics10010041/s1, items of the questionnaire used in the analysis in this paper.
