# Supplementary material for: The Use of E-Cigarettes among High School Students in Poland Is Associated with Health Locus of Control but Not with Health Literacy: A Cross-Sectional Study
Source: Toxics. 2022 Jan 17;10(1):41. doi: 10.3390/toxics10010041 (PMC8778307; doi:10.3390/toxics10010041)
Supplement: Supplementary file 1 [file toxics-10-00041-s001.zip › toxics-1554807-supplementary.pdf]

Items of the questionnaire used in the analysis presented in the article

Date __________________________

Place of survey (School, Address)

Gender

- □ female
- □ male

Age __________________________ years

Class at school __________________________ (1, 2, 3 or 4?)

Education of your mother (legal guardian)

- □ primary
- □ vocational
- □ secondary
- □ university

Education of your father (legal guardian)

- □ primary
- □ vocational
- □ secondary
- □ university

Place of residence

- □ urban above 400,000 inhabitants
- □ urban from 200,000 to 400,000 inhabitants
- □ urban from 100,000 to 200,000 inhabitants
- □ urban from 10,000 to 100,000 inhabitants
- □ urban below 10,000 inhabitants
- □ rural

What is the size of accommodation (flat/house) you live?

- □ below 30 m^2
- □ from 31 m^2 to 50 m^2
- □ from 51 m^2 to 70 m^2
- □ from 71 m^2 to 90 m^2
- □ above 90 m^2

What is your number of siblings? ___________________ persons

What is marital status of your parents/guardians?

- □ married
- □ informal
- □ divorced or in separation
- □ one of parents died
- □ both parents died

Has your family received some type of external support/help?

- □ financial
- □ material
- □ in the form of service
- □ we have not received any external help

How do you assess financial situation of your family?

- □ very good
- □ good
- □ average

□ bad
□ very bad

How much do you spend montly on your mobile phone?

□ below 5 PLN
□ 5 – 10 PLN
□ 10 – 30 PLN
□ 30 – 50 PLN
□ above 50 PLN
□ I have not got mobile phone

How many books (without textbooks and e-books) are there in at your home?

□ no book collection
□ not more than 25 books
□ 26 – 50 books
□ 51 – 100 books
□ 101 – 500 books
□ above 500 books

Do you use the Internet?

□ yes
□ no

How often do you use the Internet?

□ everyday
□ several times a week
□ once a week
□ several times monthly but not each week
□ not more often than once monthly

How many hours do you use the Internet in a week?

□ not more than 2 hours
□ more than 2 hours but not more than 7 hours
□ more than 7 hours but not more than 14 hours
□ more than 14 hours but not more than 21 hours
□ more than 21 hours but not more than 35 hours
□ more than 35 hours

Have you ever used e-cigarettes?

□ yes
□ no

When have you used e-cigarettes the last time?

□ in the last week
□ in the last month
□ in the last three months
□ in the last year
□ more than year ago

Have you ever smoked traditional cigarettes?

□ yes
□ no

When have you smoked cigarettes the last time?

□ in the last week
□ in the last month
□ in the last three months
□ in the last year
□ more than year ago

□ ponad rok temu
